# Anterior Loculated Pericardial Effusion Misinterpreted as Right Heart Dilation Suggesting Pulmonary Embolism

**DOI:** 10.5811/cpcem.2019.1.40700

**Published:** 2019-02-26

**Authors:** Sari Lahham, Emily Frisch, Mark I. Langdorf

**Affiliations:** * University of California, Irvine, Department of Emergency Medicine, Orange, California; † University of California, Irvine, School of Medicine, Irvine, California

## Abstract

We report a case of anterior loculated pericardial effusion misinterpreted on point-of-care ultrasound as a dilated right ventricle, and suggesting diagnosis of pulmonary embolism (PE), in a patient with renal failure. The compressed right ventricle from tamponade physiology appeared to be a thickened intraventricular septum. Heparin was given empirically for presumed PE. Later the same day, computed tomography of the chest showed the effusion, as did formal echocardiogram. The patient had drainage of 630 milliliters of fluid and recovered from tamponade. Loculated effusions comprise 15% of all pericardial effusions, and misdiagnosis of PE with heparin therapy could be fatal.

## INTRODUCTION

Pericardial effusion is abnormal fluid in the pericardial sac. Increased pressure impairs diastolic filling and hence cardiac output, progressing to cardiac tamponade.^1^ Pericardial effusion clinically manifests as chest pain or pressure, weakness, near syncope and shortness of breath. Causes include pericarditis, infection, retrograde aortic dissection, post-myocardial infarction free wall rupture, renal failure, malignancy, and trauma. Loculated effusions are more common when scarring has supervened (e.g., postsurgical, post-trauma, post-purulent pericarditis),^2^ and comprise 15% of effusions.^3^

Point-of-care ultrasound (POCUS) is typically readily diagnostic of pericardial effusion. The fluid appears anechoic, typically circumferential around the heart.^1^,^2^ Smaller effusions appear as a thin stripe, visible only posteriorly with gravity. The right side of the heart is most susceptible to compression by fluid since it is low pressure. Diastolic collapse of the right ventricle and right atrium defines tamponade physiology. A loculated anterior effusion has potential to cause tamponade. Although loculated pericardial effusion has been recently reported, POCUS simulating right heart strain and pulmonary embolism (PE) has not. 4

## CASE REPORT

A 46-year-old Asian man with history of hypertension, end-stage renal disease on dialysis, thrombotic stroke, and chronic tobacco use presented to the emergency department (ED) with chief complaint of weakness, lightheadedness, and shortness of breath for two days. He had dialysis one day before, but did not feel better. He developed central chest pain at rest four to five hours prior to arrival, which was worse with deep inspiration. He was seen at an outside hospital and was told he had a pericardial effusion. He was then sent to our ED for higher level of care.

On arrival, blood pressure was 124/89 millimeters of mercury (mmHg), heart rate 120 beats per minute, respiratory rate 18 per minute, oral temperature 37.4° centigrade and oxygen saturation 93% on room air. His body mass index was 23 kg/m^2^. His physical exam was notable for warm and dry skin, normal mentation, hyperdynamic precordium, normal S1 and S2, and no audible murmur, rub or gallop. There was jugular venous distention while sitting up at 90 degrees, but this was not specifically measured. There were no rales of pulmonary congestion and he had no leg edema or complaints of pain.

POCUS did not reveal circumferential or dependent effusion or tamponade physiology. The bedside image was interpreted as an enlarged right ventricle (RV), nearly twice the transverse dimension of the left ventricle, with a thickened intraventricular septum, suspicious for right heart strain (Video).

**Image 1:**
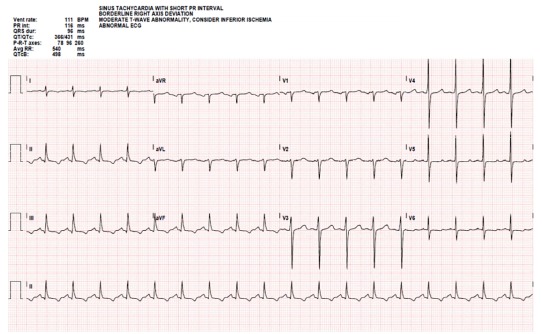
Electrocardiogram showing sinus tachycardia rate 111, but no low voltage, electrical alternans or signs of hyperkalemia.

**Image 2:**
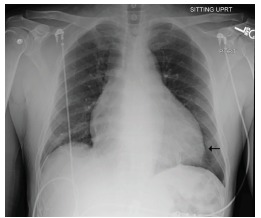
Chest radiograph demonstrating cardiomegaly (arrow) but no vascular congestion.

**Image 3:**
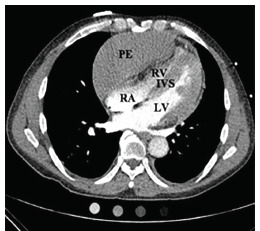
Computed tomography angiography showing loculated anterior pericardial effusion (PE), right ventricle (RV), left ventricle (LV) and intraventricular septum (IVS). There was a 6.0 x 1.3 cm pericardial fluid collection, which extended predominantly along the right heart border and superiorly along the superior pericardial recess. There was severe compression of the RV and right atrium (RA).

The patient had laboratory studies, electrocardiogram (Image 1), anterior-posterior portable chest radiograph (Image 2), and computed tomography angiography (CTA) to assess for PE (Image 3), among other diagnoses. He was given aspirin, and unfractionated heparin bolus and drip per cardiology recommendations pending CTA, which was done upon admission a few hours after presentation to the ED. He was admitted to the coronary care unit. The CTA then revealed a loculated anterior pericardial effusion, and the thickened septum was determined to be the compressed RV, which had not been appreciated on POCUS.

His initial troponin was 0.23 nanograms per milliliter (ng/mL) (normal < .03 ng/mL) in the ED, and rose to 0.26 upon admission six hours later. This was thought to be due to renal failure and not acute coronary syndrome per the inpatient team. The patient had pericardiocentesis of 630 mL sterile serosanguinous fluid under ultrasound guidance in the cardiac catheterization lab. Initial intrapericardial pressure was 20 mmHg. The cause of the effusion was ultimately attributed to uremia. The patient had no history of infection or cardiac surgery to predispose to loculation. Fortunately, there was no complication of the unnecessary anticoagulation.

**CPC-EM Capsule**

What do we already know about this clinical entity?

*Pericardial effusions are consistently well and rapidly diagnosed through point-of-care ultrasound, given standard presentation*.

What makes this presentation of disease reportable?

*A loculated pericardial effusion can simulate an enlarged right ventricle and lead to misdiagnosis of pulmonary embolism (PE)*.

What is the major learning point?

*Loculated pericardial effusions comprise 15% of all effusions, with 80% circumferential and 5% unspecified*.

How might this improve emergency medicine practice?

*Recognition of this entity can avoid anticoagulation for PE, which may worsen effusion and lead to tamponade*.

## DISCUSSION

Pericardial effusion has traditionally been diagnosed via POCUS in the ED. Typical circumferential pericardial effusions are drained percutaneously with a small catheter.^3^ However, approximately 15% of the time, effusions become loculated from adhesions.^2^ Common causes include scarring after trauma and purulent pericarditis.^2^ Identifying loculated effusion is significant, as surgical pericardiectomy drainage is preferred.^4^ This report highlights a pitfall of POCUS, but emphasizes the importance of the entire clinical presentation.

The patient’s hypoxia, tachycardia, shortness of breath and chest pain were consistent with the working diagnosis of PE, but his relatively narrow pulse pressure, enlarged cardiac silhouette and history of dialysis-dependent renal failure suggested pericardial effusion. If POCUS is not convincing in a patient with suspicion for pericardial effusion, CT imaging or formal echocardiogram should be considered to help differentiate, as in this case. Early identification of pericardial effusion can be critical in preventing and treating tamponade. We recommend POCUS, formal ultrasound or CTA of the chest to evaluate these two life threats.

## CONCLUSION

Pericardial effusions are commonly diagnosed in the ED through POCUS. If clinical suspicion suggests pericardial effusion but circumferential fluid is not seen, loculated effusion should be considered. CTA or formal ultrasound will differentiate between these two life threats.

## Supplementary Information

Video Video clip of point-of-care ultrasound.

## References

[1] Lee EK (2017). Malignant pericardial effusion presenting as a wheeze-case report. Crit Care Shock.

[2] Maisch B, Seferovic PM, Ristic AD (2004). Guidelines on the Diagnosis and Management of Pericardial Diseases, European Society of Cardiology. Eur Heart J.

[3] Kopecky SL, Callahan JA, Tajik AJ (1986). Percutaneous pericardial catheter drainage: report of 42 consecutive cases. Am J Cardiol.

[4] Tsang TS, Enriquez-Sarano M, Freeman WK (2002). Consecutive 1127 therapeutic echocardiographically guided pericardiocenteses: clinical profile, practice patterns, and outcomes spanning 21 years. Mayo Clin Proc.

[5] Yang ZM, Pontius E, Motalib S (2018). Idiopathic loculated and septated pericardial effusion. J Emerg Med.

